# JI017, a Complex Herbal Medication, Induces Apoptosis via the Nox4–PERK–CHOP Axis in Ovarian Cancer Cells

**DOI:** 10.3390/ijms222212264

**Published:** 2021-11-12

**Authors:** Taewoo Kim, Seong-Gyu Ko

**Affiliations:** Department of Preventive Medicine, College of Korean Medicine, Kyung Hee University, Seoul 130-701, Korea; tae1410@naver.com

**Keywords:** JI017, ER stress, ROS, Nox4, exosome

## Abstract

Many anti-cancer drugs, including paclitaxel and etoposide, have originated and been developed from natural products, and traditional herbal medicines have fewer adverse effects and lesser toxicity than anti-tumor reagents. Therefore, we developed a novel complex herbal medicine, JI017, which mediates endoplasmic reticulum (ER) stress and apoptosis through the Nox4–PERK–CHOP signaling pathway in ovarian cancer cells. JI017 treatment increases the expression of GRP78, ATF4, and CHOP and the phosphorylation of PERK and eIF2α via the upregulation of Nox4. Furthermore, it increases the release of intracellular reactive oxygen species (ROS), the production of intracellular Ca^2+^, and the activation of exosomal GRP78 and cell lysate GRP78. Combination treatment using the sarco/endoplasmic reticulum Ca^2+^-ATPase inhibitor thapsigargin (TG) and JI017 reportedly induces increased ER stress and cell death in comparison to the control; however, knockdown experiments of PERK and CHOP indicated suppressed apoptosis and ER stress in JI017-treated ovarian cancer cells. Furthermore, targeting Nox4 using specific siRNA and pharmacological ROS inhibitors, including N-acetylcystein and diphenylene iodonium, blocked apoptosis and ER stress in JI017-treated ovarian cancer cells. In the radioresistant ovarian cancer model, when compared to JI017 alone, JI017 co-treatment with radiation induced greater cell death and resulted in overcoming radioresistance by inhibiting epithelial–mesenchymal-transition-related phenomena such as the reduction of E-cadherin and the increase of N-cadherin, vimentin, Slug, and Snail. These findings suggest that JI017 is a powerful anti-cancer drug for ovarian cancer treatment and that its combination treatment with radiation may be a novel therapeutic strategy for radioresistant ovarian cancer.

## 1. Introduction

Ovarian cancer is the fifth most common cancer occurring in woman globally [1]. Ovarian tumors can derive from three cell types: epithelial cells, germ cells, and stromal cells [2]. Approximately 90% of ovarian tumors originate from epithelial cells, however, 2–3% from germ cells and 5–6% from stromal cells [3]. Chemotherapy is a very strong therapeutic strategy for patients with ovarian cancer [4]. Despite the development of ovarian cancer therapies, such as surgery, chemotherapy, hormone therapy, targeted therapy, immune therapy, and radiation therapy, the cancer is often chemoresistant [5]. Many studies reported that the epithelial–mesenchymal transition (EMT) causes chemoresistance on cancer therapy using anti-cancer reagents such as paclitaxel and carboplatin [6]. EMT is a driver for chemoresistance via various mechanisms such as MAPK/ERK, TGFβ-SMAD, JAK/STAT, PI3K-AKT [7]. The molecular mechanisms leading to therapy resistance by the EMT process are still not clear and require deeper insight. To overcome this chemoresistance, we need to suggest a new treatment strategy for recurrent ovarian cancer so that patients receive the most effective therapy. Herbal medicines extracted from plants may be potential anti-cancer therapeutics to overcome chemoresistance [8].

We developed and studied a novel complex herbal medication JI017 with intensive anti-tumor effects in various cancer types. JI017 is a combination of herbal extracts from Angelica gigas (Ag), Zingiber officinale Roscoe (Zo), and Aconitum carmichaeli (Ac) in the ratio of 2:1:1. The anti-cancer effects of Ag, Zo, and Ac individually have been well reported in many cancer cells, including pancreatic cancer, liver cancer, breast cancer, lung cancer, and oral cancer [9,10,11,12]. Ag ethanol extracts induce caspase-3-dependent apoptosis by inhibiting MMP-2 and MMP-9 in Human pancreatic cancer cells [9]. Zo ethanol extracts mediate caspase-3-dependent apoptosis by activating autophagic cell death and cell cycle arrest [13]. Ac extracts exert an anti-inflammatory effect, but other effects are not clear [14].

Radiotherapy is a powerful tumor therapeutic strategy used in most cases of cancer [15]. However, radioresistance can still be acquired, and this problem is a serious obstacle to cancer therapy [16]. Molecular mechanism studies to overcome radioresistance are necessary and understanding the biological functions that vary between radiosensitivity and radioresistance may suggest novel strategies for potential anti-cancer effects by overcoming radioresistance. EMT is a biological phenomenon wherein the epithelial cells lose their properties of cell-to-cell adhesion and apicobasal polarity and become mesenchymal stem cells by gaining invasive and migratory functions [17]. In tumorigenesis and cancer progression, EMT play an important role in, and contribute to, process-acquired resistance, such as radioresistance [18]. EMT is regulated by diverse signaling pathway and markers, including loss of E-cadherin and increase of N-cadherin, vimentin, Snail, Slug, transforming growth factor-β, Wnt, EGFR, PI3K, Akt, ERK, nuclear factor-kappa B, and Notch, leading to radioresistance in various cancer types [19,20]. In hematopoietic progenitor cells of wild-type mice, the upregulation of the EMT marker Slug mediates chemoresistance and survival pathway after radiation exposure; however, in hematopoietic progenitor cells of Slug^-/-^ mice, Slug knockdown induces apoptosis after radiation exposure [21]. Furthermore, downregulation of the epithelial marker E-cadherin and the upregulation of mesenchymal markers N-cadherin and vimentin are potential factors inducing radioresistance [22].

Endoplasmic reticulum (ER) stress induces cell death via the activation of unfolded protein response (UPR) in the tumor environment, and recently, targeting ER stress to overcome chemoresistance has been shown to allow for powerful and promising anti-cancer strategies, such as combinatory therapies [23]. Reactive oxygen species (ROS) contribute to many diseases, and ROS-mediated stresses regulate tumor growth and are an important factor for cell death induction via the activation of ER stress [24]. Excessive ROS mediates the programmed cell death cascade, including caspase-3, caspase-9, and PARP cleavage, in various cancer types, and it may provide a powerful anti-cancer therapy strategy [25]. The NAPDH oxidase (Nox) family, including Nox2 and Nox4, regulates ROS production [26]. Furthermore, accumulating evidence indicates redox signaling regulators, including Nox4, Ero-1, and calcium, and the relationship between ROS and ER stress plays potential roles in diverse diseases, such as cancer, inflammation, and diabetes [27]. A novel transient receptor potential vanilloid 1 antagonist, DWP05195, induces cell death and ER stress by releasing ROS in ovarian cancer cells, and Nox knockdown using p47phox siRNA blocks DWP05195-mediated CHOP induction and cell death. [28]. Traditional medicine derived compounds exert anti-cancer effects via ER stress in various cancer types [29]. Polyphyllin D derived *Paris polyphylla* induces ER stress and cell death via GRP78, CHOP, and caspase-3 cleavage in the NSCLC cell line NCI-H460; furthermore, the *Saussurea lappa* and *Aucklandia lappa derivative* dehydrocostuslactone mediates ER stress and cell death by activating PERK–CHOP and IRE-1–JNK signaling pathways and inducing ROS and Ca^2+^ release in the NSCLC cell line A549 and NCI-H460 [30,31]. Guggulsterone extracted from *Commiphora mukul* reportedly induces apoptosis via the upregulation of GRP78, PERK, p-Jun N-terminal kinase (p-JNK), CHOP, and DR5 in Hep3B cells; further, CHOP knockdown inhibited the anti-cancer effect of guggulsterone [32]. 3,3′-Diindolylmethane (DIM), a bioactive compound derived from Brassica spp., including kale and broccoli, induces apoptosis via the activation of GRP78–PERK/IRE1α–CHOP signaling pathway and the inhibition of EMT, by downregulating E-cadherin and upregulating N-cadherin, vimentin, Slug, and Snail [33]. Therefore, ER stress-apoptosis may be a potential tumor therapeutic strategy to overcome tumor progression, metastasis, invasion, and radioresistance through EMT inhibition.

Recent reports suggest that exosomes, cell-to-cell communicators, and cell-derived vesicles increase the survival ability of cancer cells during radiotherapy [34]. In contrast, exosomes released by anti-cancer drugs play a role in cell death, and therefore, therapeutic strategy using exosomes exerts potential anti-cancer effects and overcomes resistance by inhibiting EMT in the tumor environment [35]. Moreover, the activation of ER stress releases exosomes in hepatocellular carcinoma cells, and these exosomes regulate anti-cancer immunity through the inhibition of programmed death ligand 1 [36].

In the present study, we sought to examine whether JI017 mediates apoptosis via ER stress in ovarian cancer cells and whether JI017 regulates ER stress and cell death via the ROS pathway and the release of Nox. We identified that JI017 causes apoptosis via the PERK–ATF4–CHOP axis and Ca^2+^ release and induces ER stress and apoptosis by releasing Nox4 and ROS in ovarian cancer cells. Therefore, we suggest that the novel herbal medicine JI017 is a potential therapeutic for ovarian cancer.

## 2. Results

### 2.1. Anti-Ovarian Cancer Effects of JI017 In Vitro and In Vivo

**Figure 1:**
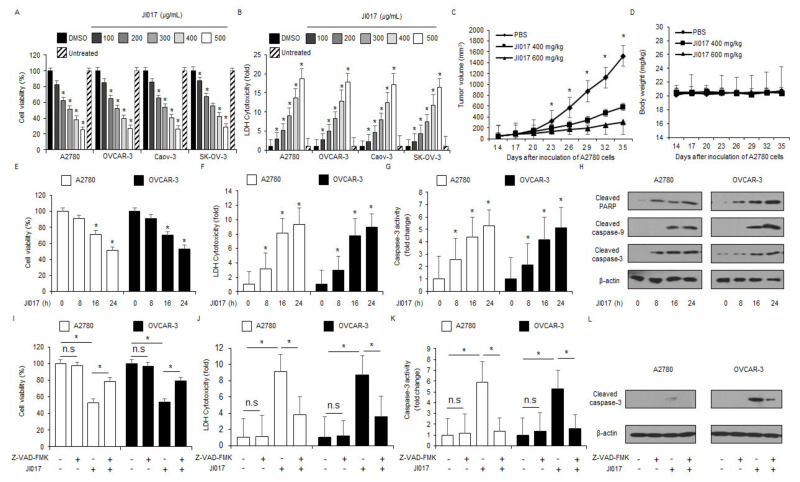
Cytotoxic effects of JI017 in ovarian cancer cell lines. (**A**,**B**) JI017 dose-dependent cell viability and LDH cytotoxicity in ovarian cancer cell lines, including A2780, OVCAR-3, Caov-3, and SK-OV-3, measured using the WST-1 assay and LDH assay on 96-well plates. (**C**,**D**) A2780 cells (1 × 10^7^) were implanted (sc) into the thigh on the right hind leg of nude mice (n = 10/group). JI017 (400 or 600 mg/kg) or 10% DMSO was administered (ip) once a day for two days. The longest (*L*) and shortest (*W*) axes of the tumors were measured, and the tumor volume (mm^3^) was calculated as LW2/2. Body weights of the A2780 tumor-xenograft mice were determined twice a week during the experiment. (**E**–**H**) JI017 treatment. (**I**–**L**) The effect of Z-VAD-FMK (50 μM) and JI017 treatment. A2780 and OVCAR-3 cells were pre-treated with Z-VAD-FMK for 4 h and were subsequently treated with JI017 (300 µg/mL, 24 h). Cell viability was determined using the WST-1 assay; cell cytotoxicity was monitored using the LDH assay, and caspase-3 activity was assessed using the caspase-3 activity assay; *, *p* < 0.05. To identify caspase-3 cleavage, Western blot analysis was performed. β-actin was used as a protein loading control.

To identify the anti-cancer effect of JI017 in ovarian cancer cell lines, including A2780, OVCAR-3, Caov-3, and SK-OV-3, we tested the cell viability and cytotoxicity using WST-1 and LDH assays in a dose-dependent manner (Figure 1A,B). JI017 treatment caused dose-dependent reduction of cell viability and increase of LDH cytotoxicity in ovarian cancer cell lines when compared to the control (Figure 1A,B). To validate the effects of JI017 in vivo, an ovarian cancer xenograft mice model was constructed using A2780 cells. Mice in the 400 and 600 mg/kg JI017 groups exhibited lower tumor volumes than those in the control group (Figure 1C). The body weights of all groups were not significant (Figure 1D). To study the anti-cancer effect of JI017 in a time-dependent manner, we tested cell viability, cell cytotoxicity, and caspase-3 activity assays using WST-1, LDH, and caspase-3 activity assay in a time-dependent manner, respectively (Figure 1E–G). JI017 treatment induced decrease in cell viability and enhancement of LDH release and caspase-3 activity in a time-dependent manner in ovarian cancer cell lines A2780 and OVCAR-3 (Figure 1E–G). Western blot analyses in a time-dependent manner revealed that JI017 induced caspase-3, caspase-9, and PARP cleavage (Figure 1H). To confirm whether JI017 regulates caspase-dependent apoptosis in ovarian cancer cells, we performed a pharmacological inhibitor experiment using the caspase inhibitor Z-VAD-FMK. Z-VAD-FMK alone did not affect cell viability, LDH cytotoxicity, and caspase-3 activity; however, JI017 alone decreased cell viability and increased LDH release and caspase-3 activity. In combination with Z-VAD-FMK, JI017 dramatically inhibited the reduction of cell viability and enhancement of LDH cytotoxicity and caspase-3 activity (Figure 1I–K). Furthermore, Western blot analyses indicated that JI017 + Z-VAD-FMK blocked caspase-3 cleavage to a greater extent than JI017 alone (Figure 1L). Our finding suggested that JI017 treatment induces apoptosis in ovarian cancer cell lines.

### 2.2. JI017 Induces ER Stress in Ovarian Cancer Cells

**Figure 2:**
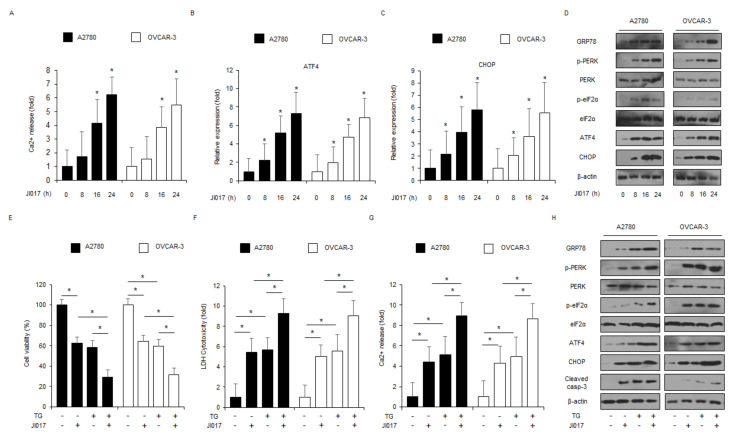
JI017 induces ER stress and cell death via intracellular Ca^2+^ release. (**A**) A2780 and OVCAR-3 cells were treated with JI017 (300 µg/mL) in a time-dependent manner (0, 8, 16, and 24 h), and intracellular Ca^2+^ release was determined using intracellular Ca^2+^ assay. (**B**,**C**) A2780 and OVCAR-3 were treated with JI017 (300 µg/mL) in a time-dependent manner (0, 8, 16, and 24 h), and mRNA levels of ATF4 and CHOP were investigated using real-time PCR. β-actin was used as a housekeeping gene. (**D**) A2780 and OVCAR-3 cells were treated with JI017 (300 µg/mL) for the indicated times (0, 8, 16, and 24 h), and the activation of ER stress signaling, including GRP78, p-PERK, PERK, p-eIF2α, eIF2α, ATF4, and CHOP, was assessed using Western blot analyses. β-actin was used as a protein loading control. (**E**–**H**) Western blot analysis of GRP78, p-PERK, PERK, p-eIF2α, eIF2α, ATF4, CHOP, and cleaved caspase-3 levels were determined using WST-1 assay, LDH assay, and intracellular Ca^2+^ assay in thapsigargin (TG; 3 μM, 24 h)- and JI017 (300 µg/mL, 24 h)-treated ovarian cancer cells; *, *p* < 0.05.

Intracellular calcium (Ca^2+^) release from ER frequently induces ER stress and cell death via protein folding, the activation of UPR and ER homeostasis [37]. To determine whether JI017 modulates Ca^2+^ release, intracellular Ca^2+^ release assay was performed. When A2780 and OVCAR-3 cells were treated with JI017 in a time-dependent manner, intracellular Ca^2+^ release increased approximately 5–6 times in comparison to the control (Figure 2A). To identify the mRNA expression of ER stress markers, including ATF4 and CHOP, in JI017-treated A2780 and OVCAR-3 cells in a time-dependent manner, we performed real-time PCR. JI017 treatment increased ATF4 and CHOP expression to a greater extent than the control treatment (Figure 2B,C). To study the protein expression of ER stress markers, such as GRP78, p-PERK, PERK, p-eIF2α, eIF2α, ATF4, and CHOP, in JI017-treated A2780 and OVCAR-3 cells in a time-dependent manner, we performed Western blot analyses. JI017 treatment enhanced the expression levels of GRP78, p-PERK, p-eIF2α, ATF4, and CHOP to a greater extent than the control treatment (Figure 2D). Many reports suggested that GRP78 regulates cell survival and cell death via cell-to-cell communication in exosome isolates [38]. To identify whether JI017 treatment induces exosomal GRP78 in A2780 and OVCAR-3 cells, we isolated the exosome from JI017-treated ovarian cancer cell culture media and performed Western blot analyses. JI017 treatment upregulated exosomal GRP78 and the exosome marker CD63 in a time-dependent manner (Supplementary Figure S1A). To further confirm whether JI017 mediates apoptosis through ER stress, we co-treated the A2780 and OVCAR-3 cells with JI017 and thapsigargin (TG), an ER stress inducer or 4-phenylbutyric acid (4-PBA), an ER stress inhibitor. TG in combination with JI017 inhibited cell viability and increased LDH and Ca^2+^ release to a greater extent in comparison with JI017 or TG alone (Figure 2E–G). TG + JI017 induced the upregulation of GRP78, p-PERK, p-eIF2α, ATF4, and CHOP and caspase-3 cleavage in A2780 and OVCAR-3 cells to a greater extent in comparison with TG or JI017 alone (Figure 2H). In combination with 4-PBA, JI017 dramatically inhibited the reduction of cell viability and enhancement of LDH cytotoxicity and Ca^2+^ release (Supplementary Figure S1B–D). Furthermore, Western blot analyses indicated that JI017 + 4-PBA blocked the upregulation of GRP78, p-PERK, p-eIF2α, ATF4, and CHOP and caspase-3 cleavage to a greater extent than JI017 alone (Supplementary Figure S1E).

### 2.3. GRP78 Loss Blocks JI017-Induced ER Stress and Apoptosis in Ovarian Cancer Cells

**Figure 3:**
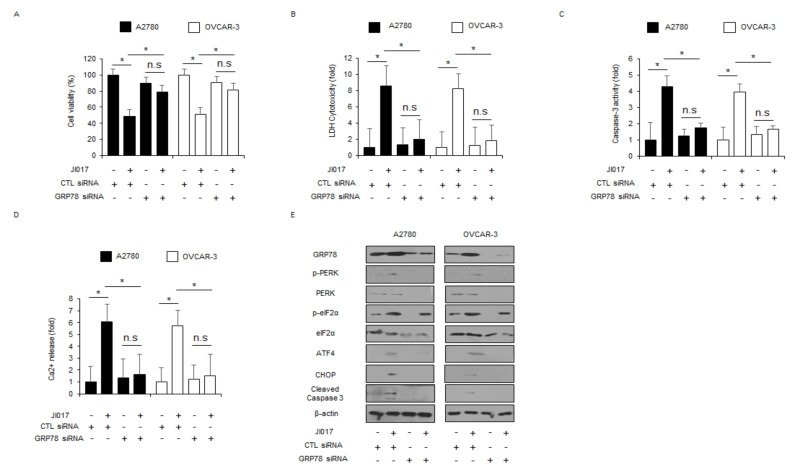
GRP78 inhibition suppresses JI017-induced ER stress and cell death in ovarian cancer cells. (**A**–**D**) A2780 and OVCAR-3 cells were transfected with GRP78-specific siRNA in the presence or absence of JI017 (300 µg/mL, 24 h), and WST-1, LDH, caspase-3 activity, and intracellular Ca^2+^ assay were performed; *, *p* < 0.05. (**E**) Western blot analysis of GRP78, p-PERK, PERK, p-eIF2α, eIF2α, ATF4, CHOP, and cleaved caspase-3 in JI017 (300 µg/mL, 24 h)-treated A2780 and OVCAR-3 cells in the presence or absence of GRP78 siRNA (30 nM, 24 h). β-actin was used as a protein loading control.

To validate if JI017 regulates the master regulator of ER stress GRP78, GRP78-specific siRNAs were transfected into A2780 and OVCAR-3 cells and then treated with JI017. A knockdown experiment using GRP78 siRNAs showed the inhibition of the reduction of cell viability and increase of LDH release, Ca^2+^ release, and caspase-3 activity in comparison to the knockdown of control siRNA in JI017-treated A2780 and OVCAR-3 cells (Figure 3A–D). Western blot analyses revealed that GRP78 loss decreased GRP78, p-PERK, PERK, p-eIF2α, eIF2α, ATF4, and CHOP expression and caspase-3 cleavage levels in JI017-treated A2780 and OVCAR-3 cells to a greater extent (Figure 3E). Further, GRP78 knockdown downregulated the expression levels of exosomal GRP78 in JI017-treated A2780 and OVCAR3 cells (Supplementary Figure S2). These results indicate that targeting GRP78 blocks ER stress and apoptosis in JI017-treated ovarian cancer cells.

### 2.4. Targeting PERK and CHOP Suppresses Apoptosis via the Inhibition of ER Stress in JI017-Treated Ovarian Cancer Cells

**Figure 4:**
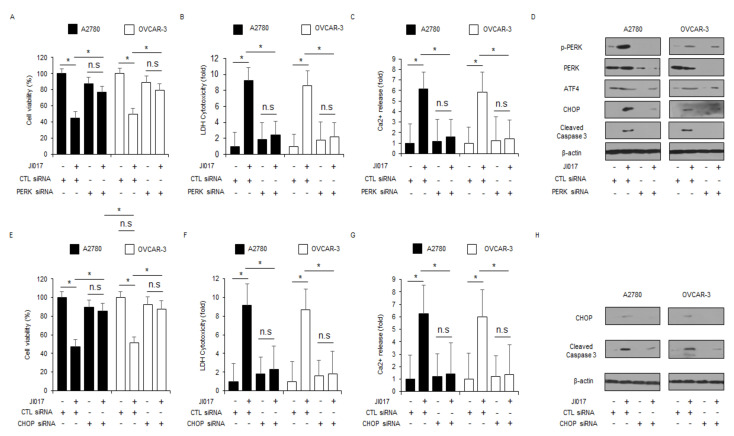
Targeting of ER stress signaling blocks JI017-induced cell death in ovarian cancer cells. (**A**–**D**) After A2780 and OVCAR-3 cells were transfected with PERK (30 nM, 24 h), the WST-1 assay, LDH cytotoxicity assay, intracellular Ca^2+^ assay, and Western blot analyses were performed with/without JI017 (300 µg/mL, 24 h) treatment.; *, *p* < 0.05. Western blot analyses were performed to identify the ER stress-related genes p-PERK, PERK, ATF4, and CHOP and caspase-3 cleavage in JI017-treated PERK knockdown cells. β-actin was used as a protein loading control. (**E**–**H**) After A2780 and OVCAR-3 cells were transfected with CHOP (30 nM, 24 h), WST-1, LDH cytotoxicity assay, intracellular Ca^2+^ assay, and Western blot analyses were performed with/without JI017 (300 µg/mL, 24 h) treatment.; *, *p* < 0.05. Western blot analyses were performed to identify the expression of CHOP and caspase-3 cleavage in JI017-treated CHOP knockdown cells. β-actin was used as a protein loading control.

To evaluate the contribution of PERK and CHOP activation in ER stress-mediated apoptosis, PERK- and CHOP-specific siRNAs were transfected into A2780 and OVCAR-3 cells, and the cells were treated with JI017. Compared with the transfection of control siRNA in JI017-treated A2780 and OVCAR-3 cells, transfection of PERK and CHOP siRNAs in these cells inhibited the reduction of cell viability and the increase of LDH and Ca^2+^ release to a greater extent (Figure 4A–C,E–G). Western blot analyses revealed that PERK knockdown decreased p-PERK, ATF4, and CHOP expression and caspase-3 cleavage levels in JI017-treated A2780 and OVCAR-3 cells to a greater extent (Figure 4D). In addition, CHOP knockdown decreased CHOP expression and caspase-3 cleavage levels in JI017-treated A2780 and OVCAR-3 cells to a greater extent (Figure 4H). These results indicate that targeting PERK and CHOP suppresses ER stress and apoptosis by JI017 treatment in ovarian cancer cells.

### 2.5. ROS Release by JI017 Treatment Induces ER Stress and Cell Death in Ovarian Cancer Cells

**Figure 5:**
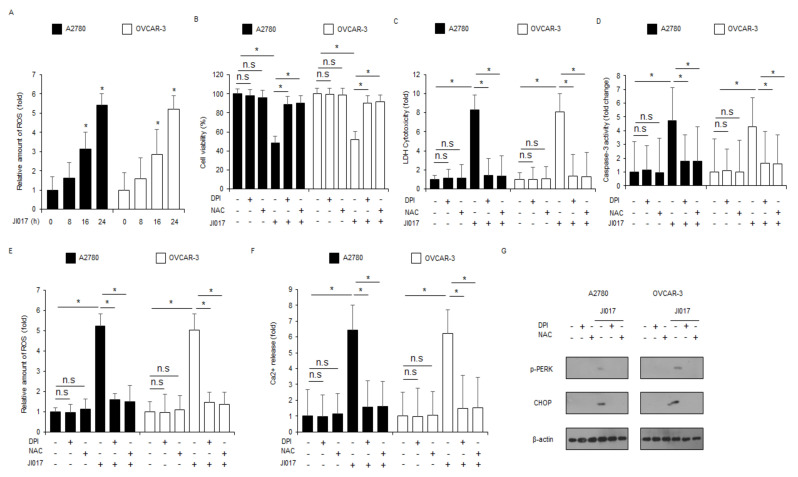
ROS inhibition by DPI or NAC blocks JI017-induced ER stress and cell death in ovarian cancer cells. (**A**) The fluorescence data indicates intracellular ROS production by DCFDA dye in JI017 (0, 8, 16, and 24 h; 300 µg/mL)-treated A2780 and OVCAR-3 cells. (**B**–**G**) A2780 and OVCAR-3 cells were pretreated with DPI (1 μM) and NAC (100 μM) for 4 h and subsequently treated with JI017 (300 µg/mL, 24 h). WST-1, LDH cytotoxicity assay, caspase-3 activity assay, ROS assay, intracellular Ca^2+^ assay, and Western blot analyses were performed; *, *p* < 0.05. (**G**) Western blot showing p-PERK and CHOP levels was performed. β-actin was used as the protein loading control.

ROS production by diverse stresses results in ER stress and apoptosis in various cancer cell types, whereas the inhibition of ROS blocks ER stress and apoptosis induced by these stressors [39]. To check for ROS release in JI017-treated ovarian cancer cells, we performed intracellular ROS assay and found that JI017 exerts intracellular ROS release in a time-dependent manner (Figure 5A). To detect whether the ROS inhibitors diphenylene iodonium (DPI) and N-acetylcysteine (NAC) suppress JI017-mediated ROS production and cell death in A2780 and OVCAR-3 cells, we performed the WST-1 assay, LDH assay, caspase-3 activity assay, intracellular Ca^2+^ assay, and intracellular ROS assay. Compared with JI017 treatment alone, DPI + JI017 or NAC + JI017 treatment inhibited the decrease of cell viability and increase of LDH release, caspase-3 activity, ROS release, and Ca^2+^ production to a greater extent (Figure 5B–F). Western blot analyses revealed that compared with JI017 treatment alone, DPI/JI017 or NAC/JI017 treatment inhibited p-PERK and CHOP expression to a greater extent, indicating the suppression of ER stress and cell death by JI017-induced ROS release (Figure 5G). These results suggest that J017 induces ER stress and apoptosis by releasing ROS in ovarian cancer cells.

### 2.6. Nox4 Is Important to JI017-Induced ROS Production and Cell Death in Ovarian Cancer Cells

**Figure 6:**
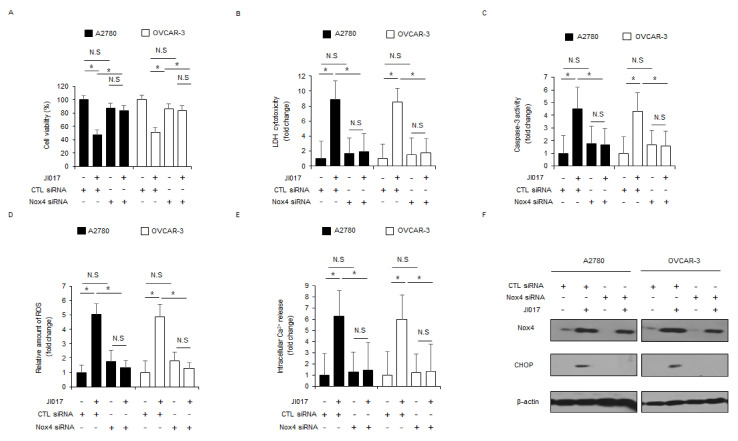
Suppression of Nox4 inhibits JI017-caused ER stress and cell death in ovarian cancer cells. (**A**–**F**) A2780 and OVCAR-3 cells were transfected with Nox4 siRNAs and treated with JI017 (300 µg/mL, 24 h). Next, WST-1 assay, LDH cytotoxicity assay, caspase-3 activity assay, intracellular ROS assay, and intracellular Ca^2+^ assay were performed along with Western blot analyses for Nox4 and CHOP; *, *p* < 0.05. β-actin was used as the protein loading control.

Silibinin, an active compound of milk thistle, induces ER stress and apoptosis by upregulating Nox4 and ROS release in prostate cancer cells [40]. To further investigate if Nox4 regulates JI017-induced ROS release, Nox4-specific siRNAs were transfected into A2780 and OVCAR-3 cells, and then the cells were treated with JI017. Compared with the experiment involving transfection of control siRNA in JI107-treated cells, knockdown experiment in JI017-treated A2780 and OVCAR-3 cells involving the transfection of Nox4 siRNAs indicated higher cell viability and lower LDH release, caspase-3 activity, intracellular ROS release, and intracellular Ca^2+^ release (Figure 6A–E). Western blot analyses revealed that compared to controls, transfection experiment involving Nox4 knockdown reduced Nox4 and CHOP levels in JI017-treated A2780 and OVCAR-3 cells to a greater extent (Figure 6F). These results indicate that targeting Nox4 blocks ER stress and apoptosis via the inhibition of Nox4 and ROS production in JI017-treated ovarian cancer cells.

### 2.7. Radiation in Combination with JI017 Overcame Radioresistance by Inhibiting EMT Phenomenon in Ovarian Cancer Cells

**Figure 7:**
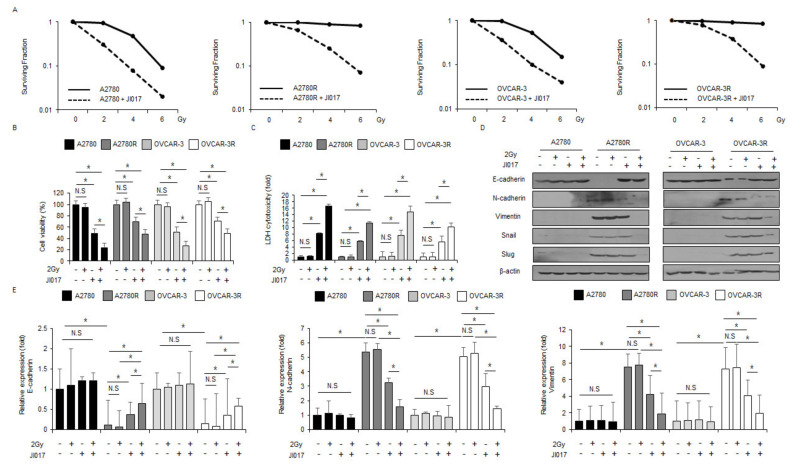
Combination of radiation and JI017 overcame radioresistance in radioresistant ovarian cancer cells exposed to radiation. (**A**) A clonogenic cell survival assay was performed at indicated doses (2, 4, or 6 Gy) of radiation, and the survival fraction was calculated using the surviving fraction formula in A2780, A2780R, OVCAR-3, and OVCAR-3R cells; *, *p* < 0.05. (**B**–**E**) A2780, A2780R, OVCAR-3, and OVCAR-3R cells were treated with JI017 (300 µg/mL, 24 h) after exposure to 2 Gy radiation. WST-1 assay and LDH assay were performed along with Western blot analyses for E-cadherin, N-cadherin, vimentin, Slug, and Snail and real-time PCR for E-cadherin, N-cadherin, and vimentin; *, *p* < 0.05. β-actin was used as the RNA and protein loading control.

Previous studies suggested that combination treatment of ionizing radiation + ER stress inducers induces apoptosis and overcomes radioresistance in cancer cells [41,42]. To identify if combination treatment using radiation with JI017 overcomes radioresistance in ovarian cancer cells, radio-resistant A2780R and OVCAR-3R cells were studied by exposing them to radiation and performing the colony formation assay, WST-1 assay, LDH assay, Western blot analysis, and real-time PCR. The results suggested that JI017 causes a decline in surviving fraction levels depending on the dose of radiation exposure (2, 4, and 6 Gy) in A2780, A2780R, OVCAR-3, and OVCAR-3R cells when compared with CTL cells (Figure 7A). JI017 diminished the cell viability and enhanced the LDH cytotoxicity in A2780 and OVCAR-3 cells to a greater extent than in A2780R and OVCAR-3R cells, and JI017/2 Gy induced lower cell viability and higher LDH cell cytotoxicity in A2780 and OVCAR-3 cells than in A2780R and OVCAR-3R cells; however, 2-Gy radiation alone showed no effect (Figure 7B,C). To probe if JI017/2 Gy suppresses EMT phenomenon in radioresistant ovarian cancer cells, we performed Western blot analyses and real-time PCR. In Western blotting analyses, no change in the expression levels of EMT markers was observed in A2780 and OVCAR-3 cells; treated with JI017 alone, 2 Gy alone, and 2 Gy/JI017 (Figure 7D). However, E-cadherin decreased, and N-cadherin, vimentin, Snail, and Slug increased in A2780R and OVCAR-3R cells (Figure 7D). Further, 2 Gy or JI017 alone resulted in almost no expression change, whereas JI017/2 Gy induced the upregulation of E-cadherin and downregulation of N-cadherin, vimentin, Snail, and Slug compared to the control (Figure 7D). Specifically, JI017/2 Gy had a powerful inhibitory effect on the EMT phenomenon to a greater extent than JI017 alone in A2780R and OVCAR-3R cells (Figure 7D). Furthermore, in real-time PCR analyses, no change in the expression levels of EMT markers was observed in A2780 and OVCAR-3 cells; treated with JI017 alone, 2 Gy alone, and 2 Gy/JI017 (Figure 7E). However, E-cadherin decreased. and N-cadherin and vimentin increased in A2780R and OVCAR-3R cells (Figure 7E). Further, 2 Gy or JI017 alone resulted in almost no expression change, whereas JI017/2 Gy induced the upregulation of E-cadherin and downregulation of N-cadherin and vimentin, compared to the control (Figure 7E). Therefore, our results indicate that JI017/2 Gy overcomes radioresistance via the inhibition of EMT phenomenon in radioresistant ovarian cancer cells.

## 3. Discussion

Many researchers have developed and studied anti-cancer therapies, including chemotherapy, radiotherapy, combination therapy, laser therapy, and surgery; however, we still face serious obstacles, such as adverse effects, drug resistance, and other challenges, while developing new technology and strategies for cancer therapy [43]. Recently, plant-derived herbal medicines have been explored with a focus on using alternative therapeutic strategy for effective anti-cancer treatment [44]. Many plants have been used for the therapy of various diseases, such as cancer, for a long time, and many researchers have investigated and identified herbal medicines having effective anti-cancer properties and milder adverse effects and toxicity than chemotherapy [45,46]. Accumulating reports suggest that herbal medicines exert potential anti-cancer effects such as apoptosis and cell cycle arrest, in various tumor types [47].

In the present study, we investigated the anti-ovarian cancer effects of novel complex herbal medication JI017 in vitro and in vivo. We demonstrated that JI017 induces apoptosis via the increase of Nox4, ROS release, caspase-3 activity, LDH cytotoxicity, Ca^2+^ release, and the decrease of cell viability in the ovarian cancer cell lines, A2780 and OVCAR-3. Furthermore, JI017 mediates ER stress and cell death by activating the PERK–eIF2α–ATF4–CHOP signaling pathway in ovarian cancer cells, and combined treatment of radiation and JI017 overcomes radioresistance by inhibiting EMT phenomena, such as the reduction of E-cadherin and the increase of N-cadherin, vimentin, Snail, and Slug in radioresistant ovarian cancer cells.

Several reports have indicated that many herbal medicines exert anti-cancer and cytotoxicity effects by activating a severe ER stress pathway in various cancers [48]. In the emergence of unfolded protein response (UPR) by the normal ER stress, the UPR plays a protective or survival role by getting rid of misfolded or unfolded proteins; however, prolonged or excessive ER stress induces apoptosis via the activation of UPR sensors, such as PERK, IRE1α, and ATF6 [49]. Polyphyllin D, a potent cytotoxic saponin isolated from *Paris polyphylla*, induces apoptosis via the GRP78–CHOP pathway in NCI-H460 cells [30]. Dehydrocostuslactone, a sesquiterpene lactone extracted from *Saussurea lappa* and *Aucklandia lappa*, activates PERK–CHOP and IRE1α–JNK–CHOP signaling pathways by releasing intracellular ROS and intracellular Ca^2+^ in NSCLC, A549, and NCI-H460 cells, leading to apoptosis [31]. Our results indicate that JI017 mediates apoptosis and excessive ER stress via intracellular ROS and Ca^2+^ production in A2780 and OVCAR-3 cells, and JI017 treatment contributes to caspase-3 and caspase-9 cleavage via the PERK–eIF2α–ATF4–CHOP signaling pathway. Combination treatment of the ER stress inducer TG + JI017 induces synergistic apoptosis, ER stress, caspase-3 activity, LDH cytotoxicity, ROS production, and Ca^2+^ release and thereby increases the phosphorylation of PERK and eIF2α and the expression of ATF4, CHOP, and caspase-3 cleavage. In contrast, targeting GRP78, PERK, and CHOP inhibits apoptosis and ER stress in JI017-treated ovarian cancer cells.

Recent reports have revealed that prolonged cellular events, such as severe ROS and Ca^2+^ release, have anti-cancer effects and cell death signaling via ER stress in tumor cells [50]. NADPH oxidases of the Nox family are potential components of ROS [51]. Many studies suggest that the activation of Nox4 is localized predominantly in the ER, and it excessively generates intracellular ROS and then causes apoptosis and ER stress [52]. Two isoforms of the Nox family, Nox2 and Nox4, are involved in apoptosis and ER stress via ROS release [53]. The inhibition of Nox4 by Nox4-specific siRNAs and the Nox4 inhibitor DPI block apoptosis and ER stress by preventing the expression of the ER stress proapoptotic marker CHOP, c-JNK, and apoptosis signal regulating kinase 1 (ASK1) [54]. In the present study, JI017 was found to cause ER stress-related apoptosis by activating Nox4-driven ROS formation in ovarian cancer cells A2780 and OVCAR-3 cells. In JI017-treated A2780 and OVCAR-3 cells, Nox4 inhibition blocked the reduction of cell viability and increased the release of LDH, caspase-3 activity, ROS production, Ca^2+^ release, as well as and ER stress responses, such as the induction of the ER stress-related proapoptotic marker CHOP. These results suggest that JI017 causes ER stress and apoptosis via the Nox4-mediated ROS production

EMT is a biological process that results in increased invasion and migration and leads to resistance, and EMT inducers, such as EMT transcription factor and EMT activator, lead to tumorigenesis and chemoresistance in various cancer types [55,56,57]. Many reports have suggested that clinical cancer radiotherapy frequently results in the EMT phenomenon in surviving cancer cells on ionizing radiation exposure, and radioresistance development is still a serious obstacle for radiotherapy [58]. Recent reports suggested that prolonged ER stress induced by a new drug inhibits the EMT process via the activation of UPR, and it may be a crucial tumor therapeutic strategy and pre-clinical model [59]. Marine, a herbal medicine derived from *Sophora flavescens*, induces anti-prostate cancer effects by activating GRP78, CHOP, and ATF4, phosphorylating eIF2α, and inhibiting the EMT process via the decrease of E-cadherin and the increase of N-cadherin and vimentin [60]. Hydroxypropyl-β-cyclodextrin, a cholesterol-depleting agent of lipid rafts, interferes with the EMT process via ER stress in breast cancer cell lines [61]. Nitidine chloride, a natural compound extracted from the root of *Zanthoxylum nitidum*, exerts powerful anti-glioma effect in vitro and in vivo via the inhibition of EMT markers, including N-cadherin, vimentin, and Slug, and the phosphorylation of the ER stress marker eIF2α [62]. Therefore, in a radioresistant tumor environment, targeting EMT phenomena could be a potential tumor therapeutic strategy to overcome radioresistance. Our results showed that 2 Gy/JI017 overcomes radioresistance and induces cell death via the upregulation of E-cadherin and the downregulation of N-cadherin, vimentin, Slug, and Snail.

**Figure 8:**
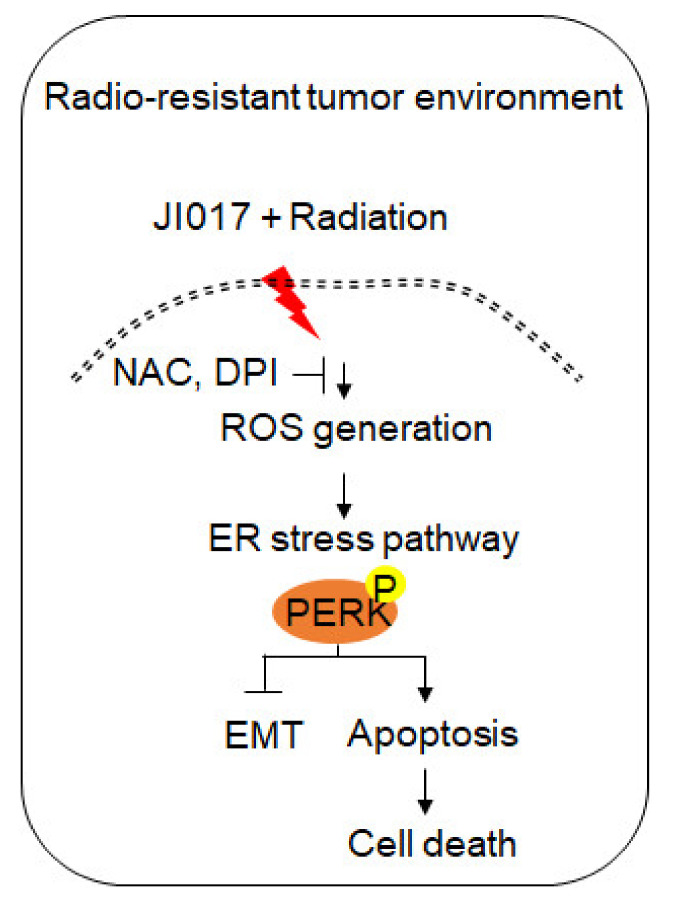
Schematic representation of ER stress and cell death pathways stimulated by JI017 and radiation in ovarian cancer cells and radio-resistant ovarian cancer cells.

In conclusion, the novel complex herbal medication JI017 induces apoptosis via Nox4–ROS–Ca^2+^–ER stress in ovarian cancer cells, and a combination therapy of radiation + JI017 overcomes radioresistance and induces apoptosis via the inhibition of EMT in radioresistant ovarian cancer cells. Our findings suggest a novel tumor therapeutic approach in tumor radiotherapy (Figure 8). However, the mechanism of anti-cancer effect in JI017-treated various cancer cell types is unclear. Further studies in future directions are needed to understand the mechanisms of JI017 in various cancer types.

## 4. Materials and Methods

### 4.1. JI017 Extraction

The complex herbal formula of JI017 was originally designed for cancer therapy, and the three ingredients were as follows: *Astragalus gigas (Ag)*, Zingiber officinale (Zo), and processed *Angelica carmichaeli (Ac)*. The mixtures were provided by Jaseng Hospital of Korean medicine (Seoul, Republic of Korea). The ingredients were mixed together, soaked in 70% ethanol, and extracted by treatment at 80 °C for 3 h, and the extract was filtered, evaporated, and lyophilized to make the JI017 powder. This was stored at −80 °C until use. 

### 4.2. Reagents

Compounds were obtained as follows: DPI (Nox or ROS inhibitor, Sigma Aldrich, St. Louis, MO, USA), NAC (a ROS inhibitor, Sigma Aldrich, St. Louis, MO, USA), Z-VAD-FMK (caspase inhibitor, Sigma Aldrich, St. Louis, MO, USA), 4-PBA (ER stress inhibitor, Sigma Aldrich, St. Louis, MO, USA), and TG (ER stress inducer, Millipore, Bedford, MA, USA).

### 4.3. Cell Culture

A2780 and OVCAR-3 human ovarian cancer cell lines were obtained from the American Type Culture Collection (Manassas, VA, USA). A2780 and OVCAR-3 cells were cultured in DMEM (Welgene, Daegu, South Korea) supplemented with 10% fetal bovine serum (FBS), 100 U/mL penicillin, and 100 mg/mL streptomycin (all from Welgene) and incubated at 37 °C under a humidified 95%/5% (*v*/*v*) mixture of air and CO_2_. To validate the phenotypic characteristics of the ovarian cancer cell lines A2780 and OVCAR-3, we performed Western blotting analyses and identified EpCAM expression (Supplementary Figure S3).

### 4.4. Cell Viability

A2780 and OVCAR-3 human ovarian cancer cell lines were seeded into a 96-well plate with DMEM medium and grown for 24 h. Then, cells were treated with JI017 for 24 h. Cell viability was tested using the WST-1 assay (Roche, Indianapolis, IN, USA) according to the manufacturer’s protocols. Cell absorbance was measured at 450 nm using an enzyme-linked immunosorbent assay reader (SpectraMax190, Microplate Reader, Molecular Devices, San Jose, CA, USA).

### 4.5. LDH Assay

A2780 and OVCAR-3 human ovarian cancer cell lines were seeded into a 96-well plate with growth medium and grown for 24 h. Then, cells were treated with JI017 for 24 h. LDH cytotoxicity assay was performed according to the manufacturer’s protocols. The fluorescence was determined by measuring the absorbance of the samples at 490 nm using the ELISA reader (SpectraMax190, Microplate Reader, Molecular Devices, San Jose, CA, USA).

### 4.6. Caspase-3 Activity Assay

A2780 and OVCAR-3 human ovarian cancer cell lines were seeded into a 6-well plate with growth medium and grown for 24 h. Caspase-3 activity assay (the Biovision colorimetric caspase-3 assay kit) was performed according to the manufacturer’s protocols. The fluorescence was measured at 405 nm using a spectrophotometer (Molecular Devices, San Jose, CA, USA).

### 4.7. Intracellular Calcium Assays

A2780 and OVCAR-3 human ovarian cancer cell lines were seeded into a 96-well plate with growth medium, and the cells were treated with JI017. Intracellular calcium assays were performed using a calcium assay kit (colorimetric; Abcam; ab102505) as described in the supplier’s manual. The fluorescence was measured and analyzed by the absorbance of the samples at 575 nm using a microplate reader (Molecular Devices, San Jose, CA, USA).

### 4.8. Intracellular ROS Assays

A2780 and OVCAR-3 human ovarian cancer cell lines were seeded and incubated into a 96-well plate with growth medium. Cells were treated with JI017 and incubated with the cell permeant 2’7’-dichlorodihydrofluorescein diacetate (Invitrogen, Loughborough, UK) for 30 min at 37 °C as described in the supplier’s protocol. The fluorescence was measured and analyzed by the absorbance of the samples at 495 nm (Ex)/525 nm (Em) using a microplate reader (Molecular Devices, San Jose, CA, USA).

### 4.9. Establishment of Radioresistant A2780 and OVCAR-3 Cell Lines

A2780 and OVCAR-3 human ovarian cancer cell lines were seeded into 60 mm dishes. To generate parental cell lines, these cells were exposed to a dose of 4 Gy daily for 12 weeks after 24 h. For establishing radioresistant A2780R and OVCAR-3 cell lines, candidates for radioresistant cells were verified by comparing with parental cells.

### 4.10. Irradiation

Human ovarian cancer cells and radioresistant human ovarian cancer cells (A2780, A2780R, OVCAR-3, and OVCAR-3R) were seeded onto 60 mm dishes with growth medium and incubated at 37 °C CO_2_ for 24 h. Then, they were subjected to ionizing radiation exposure using ^137^Cs source irradiation (Atomic Energy of Canada, Ltd., Mississauga, ON, Canada). After establishing radioresistant A2780R and OVCAR-3R cell lines subjected to a 4 Gy dose for 90 days, the established cells were grown in a growth medium containing 10% FBS.

### 4.11. Colony Formation Assay

Human ovarian cancer cells and radioresistant human ovarian cancer cells (A2780, A2780R, OVCAR-3, and OVCAR-3R) were seeded onto 60 mm dishes with growth medium and grown for 24 h at 37 °C in a CO_2_ incubator. Cells were incubated for 10 days for colony formation, and then, the colonies were stained with 0.5% crystal violet (Amresco, Solon, OH, USA). To calculate the survival fraction, the number of colonies formed was divided by the number of seeded cells in the control plate.

### 4.12. Transfection

A2780 and OVCAR-3 human ovarian cancer cell lines were transfected with double-stranded siRNAs (30 nmol/mL), such as GRP78 (Santacruz, Dallas, TX, USA), PERK (Santacruz), CHOP (Bioneer, Oakland, CA, USA), and Nox4 (Santacruz), for 24 h using Lipofectamine 2000 reagent (Invitrogen) in a 6-well plate according to the manufacturer’s protocol. 

### 4.13. Isolation of Total RNA and Protein

Total RNA from human ovarian cancer cell lines A2780 and OVCAR-3 was prepared from a 100 mm cell culture dish using Trizol reagent according to the manufacturer’s instructions (Invitrogen). Proteins from the cell lysates were collected by radioimmunoprecipitation assay lysis buffer (Bio-rad, Watford, UK). The supernatant was analyzed to quantify the proteins using the BCA method (Thermo Scientific, Waltham, MA, USA).

### 4.14. Real-Time PCR and Western Blot Analyses

Real-time PCR was performed in triplicate using an ABI Power SYBR green PCR Master Mix (Applied Biosystems, Waltham, UK) with E-cadherin-specific primers (5′- GAACGCATTGCCACATACAC-3′ (sense) and 5′-GAATTCGGGCTTGTTGTCAT-3′ (antisense)), N-cadherin-specific primers (5′-GGCATACACCATG CCATCTT-3′ (sense) and 5′-GTGCATGAAGGACAGCCTCT-3′ (antisense)), vimentin-specific primers (5′-GAGAACTTTGCCGTTGAAGC-3′ (sense) and 5′-GCTTCCTGTAGGTGGCAATC-3′ (antisense)), ATF4-specific primers ((5′-AAGCCTAGGTCTCTTAGATG-3′ (sense) and 5′- TTCCAGGTCATCTATACCCA-3′ (antisense)), and CHOP-specific primers ((5′- ATGAGGACCTGCAAGAGGTCC-3′ (sense) and 5′-TCCTCCTCAGTCAGCCAAGC-3′ (antisense)) on a Roche LightCycler 96 System (Roche). RNA quantities were normalized with β-actin primers (5′-AAGGCCAAC CGCGAGAAGAT-3′ (sense) and 5′-TGATGACCTGGCCGTCAGG-3′ (antisense)), and gene expression analyses were quantified according to the 2^−ΔCt^ method. For the Western blot analysis, cells were solubilized in RIPA lysis buffer (Bio-rad). Then, the blocked membranes were incubated overnight at 4 °C with primary antibodies. The primary antibodies used included β-actin (Santa Cruz, 1:1000, sc-47778), eIF2α (Santa Cruz, 1:1000, sc-133132), GRP78 (Santa Cruz, 1:1000, sc-166490); CD63 (Abcam, Cambridge, UK, 1:1000, ab216130); Nox4 (proteintech, 1:1000, 14347-1-AP); cleaved caspase-3 (Cell Signaling, Danvers, MA, USA, 1:1000, #9664), cleaved caspase-9 (Cell Signaling, 1:1000, #20750), cleaved PARP (Cell Signaling, 1:1000, #5625), p-PERK(Thr980) (Cell Signaling, 1:1000, #3179), PERK (Cell Signaling, 1:1000, #5683), p-eIF2α (Ser51) (Cell Signaling, 1:1000, #3398), ATF4 (Cell Signaling, 1:1000, #11815), CHOP (Cell Signaling, 1:1000, #2895), E-cadherin (Cell Signaling, 1:1000, #14472), N-cadherin (Cell Signaling, 1:1000, #13116), Slug (Cell Signaling, 1:1000, #9585), Snail (Cell Signaling, 1:1000, #3879), and vimentin (Cell Signaling, 1:1000, #5741). After washing, the membrane was incubated for 40 min at room temperature with a 1:4000 dilution of HRP-conjugated secondary antibodies. The secondary antibodies used included anti-mouse anti rabbit IgG HRP-linked antibody (Santa Cruz, sc-2357) and m-IgGK BP-HRP-linked antibody (Santa Cruz, sc-516102). The membranes were analyzed using ECL Prime Western Blotting Detection Reagents (Amersham, UK).

### 4.15. Exosome Isolation

A2780 and OVCAR-3 cells were treated with JI017 at the dose shown, and exosomes were obtained from the supernatant of JI017-treated A2780 and OVCAR-3 cells according to the manufacturer’s protocol (Total Exosome Isolation Reagent (for cell culture media), Thermo Fisher Scientific, Waltham, MA, USA). Protein concentration was measured using the BCA method (Thermo Scientific, Waltham, MA, USA). The protein loading samples (10 μg) were also quantified by Ponceau S staining and were subjected to Western blotting. Positive exosomes were identified using the exosome marker CD63.

### 4.16. Animals

For animal study, five-week-old, female, athymic BALB/c nude mice (*nu*/*nu*) were purchased from OrientBio, Inc. (Daejeon, Korea) and maintained for 1 week with free access to sterile standard mouse chow (NIH-7 open formula) and water before use. Mice were housed randomly at 50% ± 20% humidity and approximately 21 ± 2 °C on a 12 h light–dark cycle (*n* = 5 mice/group). All animal experimental procedures were performed according to the National Institutes of Health guidelines and a protocol approved by the Institutional Animal Care and Use Committee of Kyung Hee University.

### 4.17. Tumor Xenograft Mouse Models

For the mouse xenograft experiment, six-week-old mice were inoculated with a A2780 human ovarian cancer cell line by subcutaneously (sc) implanting 1 × 10^7^ cultured cells into the right thigh. Six days later, mice were grouped randomly (n = 10 per group) and JI017 (400 or 600 mg/kg) was orally administered once a day for two days. Tumor sizes on two axes (*L*, longest axis; *W*, shortest axis) were measured three times per week using Vernier calipers. Tumor volume was calculated using the formula (*L* × *W*^2^)/2 (mm^3^).

### 4.18. Statistical Analysis

All results were confirmed via at least three independent experiments; Student’s *t*-test was used to compare the means of quantitative data between groups, and a *p* value < 0.05 was considered statistically significant.

## Data Availability

Not applicable.

## Supplementary Materials

The following are available online at https://www.mdpi.com/article/10.3390/ijms222212264/s1, Figures S1–S3.
