# Supplementary material for: JI017, a Complex Herbal Medication, Induces Apoptosis via the Nox4–PERK–CHOP Axis in Ovarian Cancer Cells
Source: Int J Mol Sci. 2021 Nov 12;22(22):12264. doi: 10.3390/ijms222212264 (PMC8621090; doi:10.3390/ijms222212264)

Supplementary materials: JI017, a complex herbal medication, induces apoptosis via the Nox4–PERK–CHOP axis in ovarian cancer cells

Tae Woo Kim and Seong-Gyu Ko

Figure S1. JI017 induces ER stress in ovarian cancer cells.

(A) A2780 and OVCAR-3 cells were treated with JI017 (300 μg/mL) for the indicated times (0, 8, 16, and 24 h) and exosomes were extracted from the cell culture media. Protein samples extracted from cell lysates and exosomes were quantified by Ponceau S staining. These samples were checked by Western blot analysis using the ER stress marker, GRP78 and the exosome marker, CD63. (B-E) Western blot analysis of GRP78, p-PERK, PERK, p-eIF2α, eIF2α, ATF4, CHOP, and cleaved caspase-3 levels were determined using WST-1 assay, LDH assay, and intracellular Ca^{2+} assay in 4-PBA(10 mM, 24 h)- and JI017 (300 μg/mL, 24 h)-treated ovarian cancer cells; *, $p < 0.05$. β-actin was used as a protein loading control.

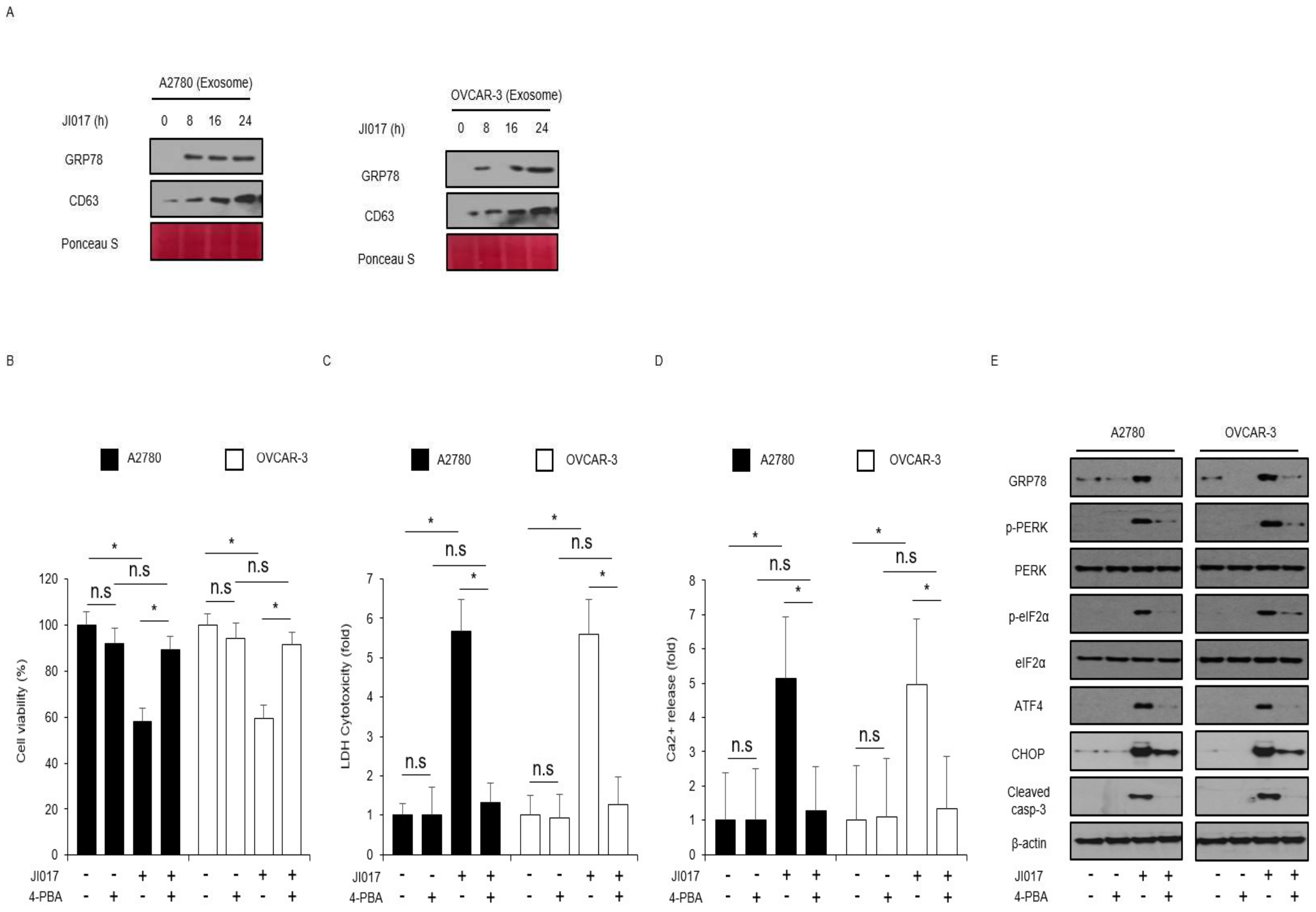

Figure S2. GRP78 inhibition suppresses JI017-induced ER stress and cell death in ovarian cancer cells.

Western blot analysis of GRP78 and CD63 in exosomes extracted from JI017 (300 μg/mL, 24 h)-treated A2780 and OVCAR-3 cell culture media in the presence or absence of GRP78 siRNA (30 nM, 24 h). Ponceau S were used as protein loading controls.

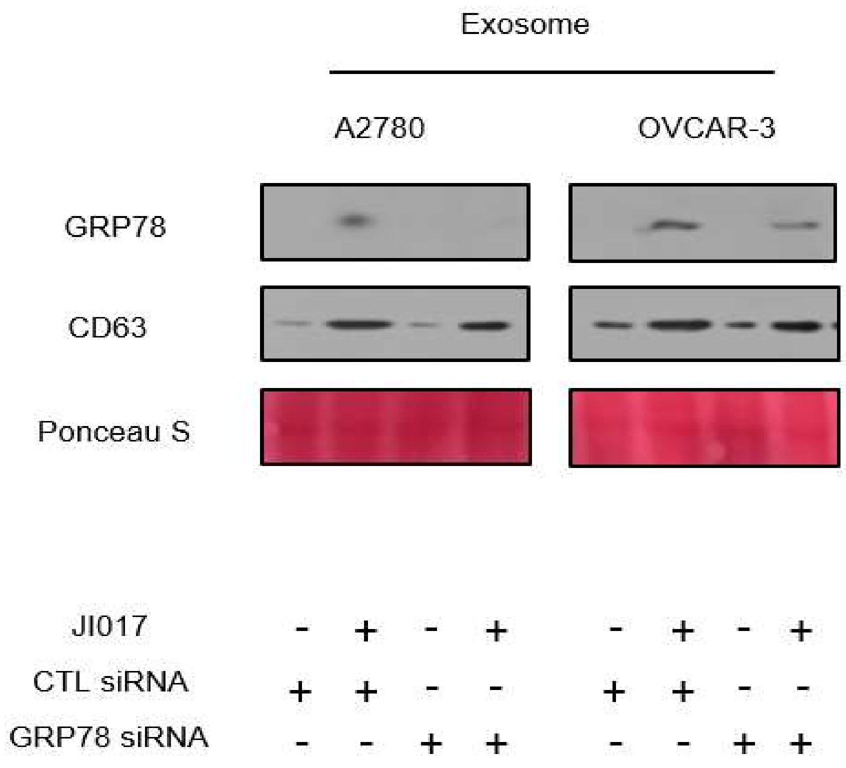

Figure S3. The phenotypic characteristics of the ovarian cancer cell lines.

Western blot analysis of EpCAM in A2780 and OVCAR-3 cell lines. . β-actin was used as a protein loading control.

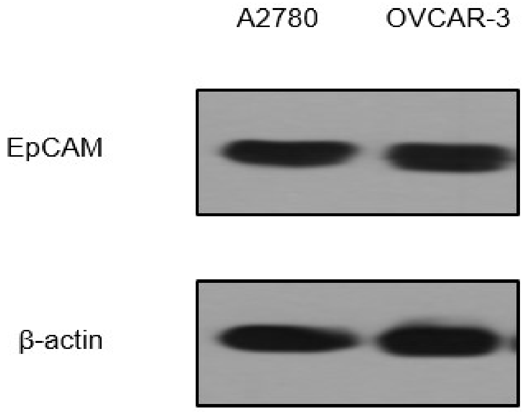

Supplement: Supplementary file 1 [file ijms-22-12264-s001.zip › ijms-1334412-supplementary.pdf]
